# The GPR183-oxysterol axis in cancer: immune trafficking, spatial organization, and therapeutic potential

**DOI:** 10.3389/fimmu.2026.1931992

**Published:** 2026-09-16

**Authors:** Haihui Zhuang, Fenglin Li, Shuangyue Li, Xia Jiang, Wenjie Sha, Renzhi Pei, Dong Chen, Ying Lu

**Affiliations:** Department of Hematology, The Affiliated People’s Hospital of Ningbo University, Ningbo, China

**Keywords:** 7α,25-OHC, CAR-T cell engineering, GPR183, oxysterols, tumor microenvironment

## Abstract

G protein-coupled receptor 183 (GPR183; also known as EBI2, Epstein-Barr virus-induced G-protein-coupled receptor 2) has emerged as a central regulator linking 7α,25-dihydroxycholesterol (7α,25-OHC) gradients to immune cell trafficking and tissue organization. In this review, we synthesize the structural, physiological, and oncogenic dimensions of the GPR183 axis. We first discuss the enzymatic biogenesis of its oxysterol ligand and the structural mechanisms underlying receptor activation. We then examine the context-dependent roles of GPR183 within the tumor microenvironment (TME), including receptor antagonism in hematological malignancies, modulation of immune organization in immunologically cold solid tumors. We further review the emerging relevance of spatial GPR183 profiling in relation to immune checkpoint blockade (ICB) responsiveness, as well as recent synthetic immunology strategies aimed at enhancing chimeric antigen receptor T (CAR-T) cell trafficking through oxysterol sensing. We identify and systematically analyze the central paradox of the GPR183 axis — namely, that identical 7α,25-OHC gradients can drive either anti-tumor tertiary lymphoid structure (TLS) formation or pro-tumor immunosuppressive myeloid cell recruitment — and discuss the potential determinants of this contextual polarity, including ligand concentration thresholds, spatial compartmentalization of oxysterol production, and receptor co-expression patterns. Collectively, current advances in the GPR183-oxysterol axis delineate new opportunities for metabolism-oriented immunotherapy and precision oncology.

## 1 Introduction

The spatial architecture and organization of immune cell populations constitute a primary determinant of host defense, a process in which G protein-coupled receptor 183 (GPR183; also known as EBI2, Epstein-Barr virus-induced G-protein-coupled receptor 2) functions as a key regulator by directing lymphocyte positioning within specialized lymphoid tissues (1, 2). Activation of this receptor depends strictly on the presence of 7α,25-dihydroxycholesterol (7α,25-OHC), an oxysterol metabolite synthesized through a coordinated enzymatic relay involving cholesterol 25-hydroxylase (CH25H) and cytochrome P450 family 7 subfamily B member 1 (CYP7B1, also termed oxysterol 7α-hydroxylase) (3, 4). The GPR183-oxysterol axis guides B cells toward the follicular mantle while simultaneously mediating the localization of dendritic cells (DCs) and T follicular helper (Tfh) cells (5, 6). Although early investigations established GPR183 as a physiological navigation system (7), contemporary evidence indicates that axis dysregulation is linked to chronic inflammatory pathologies. This mechanism is demonstrated in inflammatory bowel disease (IBD), where dysregulated accumulation of GPR183-expressing cells within the gut mucosa drives ectopic lymphoid folliculogenesis (7, 8). The systemic landscape of these physiological and pathological GPR183 functions — spanning lymph node organization, bone marrow mobilization, and TME remodeling — is depicted in **Figure 1**.

**Figure 1:**
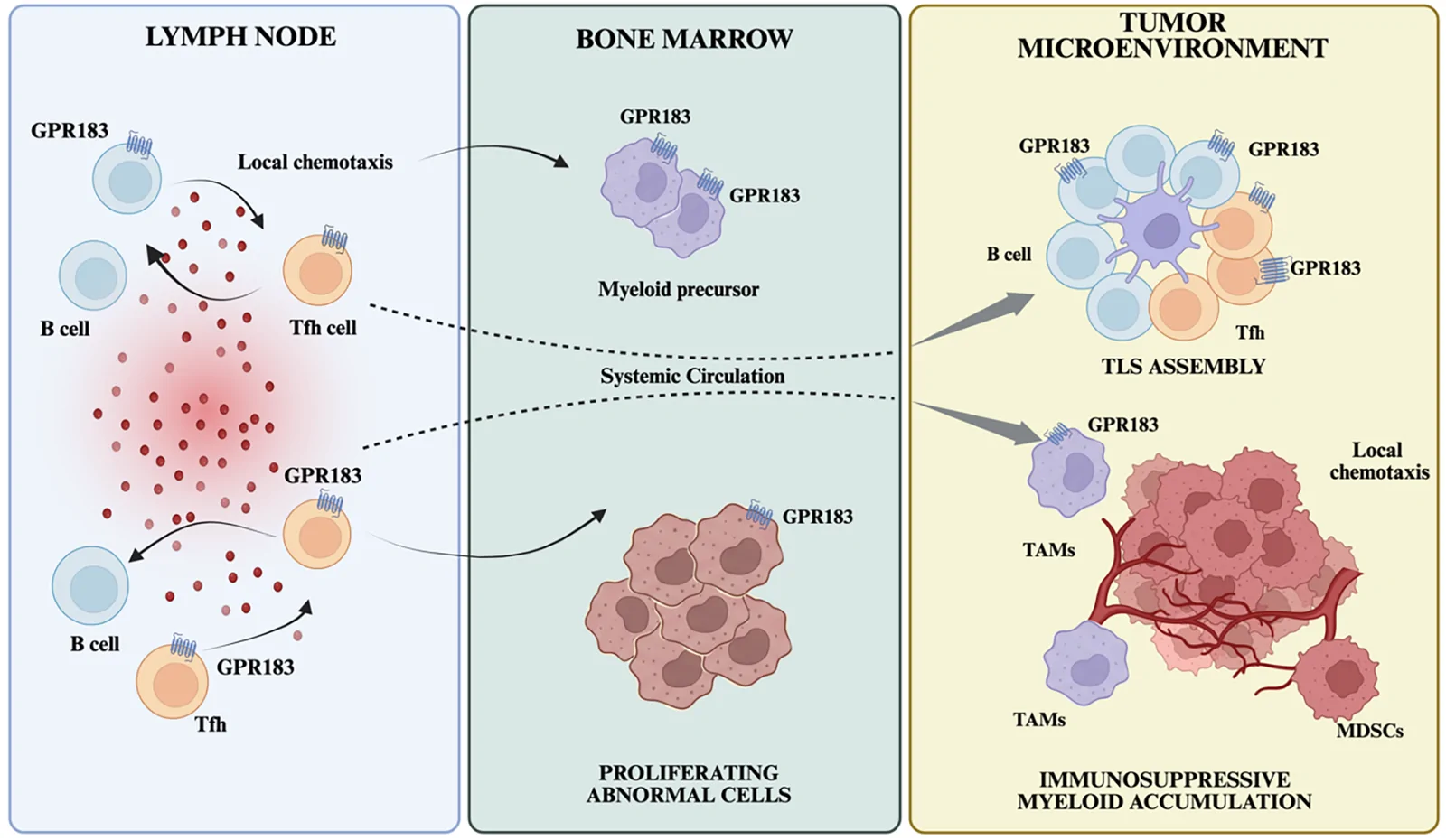
Systemic landscape of the GPR183-oxysterol axis across physiological, hematological, and solid tumor microenvironments. (Left) Lymph node: 7α,25-OHC gradients direct GPR183-mediated chemotaxis of B cells and Tfh cells, positioning lymphocytes within follicles and germinal centers. (Center) Bone marrow: GPR183 signaling drives myeloid precursor egress into the systemic circulation. Aberrant GPR183 activation in proliferating abnormal cells contributes to hematological malignancy. (Right) Tumor microenvironment: GPR183-expressing B cells and Tfh cells are recruited toward TLS assembly, while GPR183^+^ monocytes and MDSCs are recruited toward TAM-rich regions, establishing an immunosuppressive myeloid niche. Dashed arrows, systemic circulation; solid arrows, local chemotaxis. 7α,25-OHC, 7α,25-dihydroxycholesterol; CH25H, cholesterol 25-hydroxylase; CYP7B1, cytochrome P450 family 7 subfamily B member 1; DC, dendritic cell; FRC, fibroblastic reticular cell; GPR183, G protein-coupled receptor 183; HSD3B7, 3β-hydroxysteroid dehydrogenase type 7; TAM, tumor-associated macrophage; Tfh, T follicular helper cell; TLS, tertiary lymphoid structure; TME, tumor microenvironment.

Paralleling these immunological findings, systemic lipid metabolism disorders have been shown to amplify this inflammatory axis (9). Metabolic dysfunction-associated steatotic liver disease (MASLD) represents a metabolic-inflammatory spectrum within this context (9); oxysterol accumulation has been implicated in inflammatory progression during MASLD (10). Persistent chronic inflammation of this nature predisposes tissues to malignant transformation, as evidenced by the development of hepatocellular carcinoma (HCC) within this metabolic and inflammatory milieu (11). However, whether the GPR183 axis directly contributes to MASH-related hepatocarcinogenesis remains to be investigated experimentally; current evidence establishes an associative rather than a causal relationship. Thus, the GPR183 pathway serves as a biochemical bridge connecting systemic lipid dysregulation with localized chronic inflammation, though the functional role of the receptor in inflammation-driven malignant transformation requires further elucidation.

The TME co-opts this physiological navigational framework by utilizing oxysterol-mediated chemoattraction to reshape the host immune landscape. Macrophages produce oxysterols through CH25H activity (12), establishing a pathological 7α,25-OHC gradient that solid tumors utilize to facilitate immune evasion through the recruitment of tumor-promoting myeloid cells that support angiogenesis and immune suppression (13). In contrast to these extrinsic recruitment strategies, hematological malignancies such as acute myeloid leukemia (AML) exhibit distinct intrinsic oncogenic dependencies, in which leukemia blasts frequently overexpress GPR183 — a finding that correlates with poor clinical outcomes (14, 15). Conversely, in non-small cell lung cancer (NSCLC), GPR183 signaling presents an opposing functional role by inhibiting visceral metastasis (16). Addressing the physical barriers imposed by the solid tumor stroma, which typically exclude conventional adoptive cell therapies, recent synthetic biology approaches have focused on rewiring GPR183 to sense tumoral metabolites (17). Emerging synthetic immunology strategies have proposed engineering chimeric antigen receptor T (CAR-T) or natural killer (NK) cells with oxysterol-sensing properties to enhance intra-tumoral trafficking (17, 18). This review provides a synthesis of the GPR183 axis in oncology, delineating its trajectory from metabolic inflammation to the frontiers of synthetic immunology.

## 2 The biochemical foundation: the oxysterol-GPR183 signaling axis

### 2.1 The enzymatic cascade: CH25H and CYP7B1 as gradient architects

The synthesis of 7α,25-OHC depends on a stringent bimodal enzymatic relay, initiated by CH25H, which hydroxylates cholesterol at the C25 position to yield 25-hydroxycholesterol (25-HC) (19, 20). This intermediate is subsequently processed by CYP7B1, which mediates 7α-hydroxylation to generate the biologically active ligand (4), as illustrated in the upper panel of **Figure 2**. The spatial and temporal synthesis of this gradient is orchestrated by transcriptional networks in which type I interferons (IFN-α/β) trigger rapid CH25H induction in immune cells during acute inflammatory responses (21). Within the TME, dysregulated cholesterol metabolism contributes to sustained oxysterol production (22). Recent work has demonstrated that lymphatic endothelial cell-derived 25-hydroxycholesterol reprograms tumor-associated macrophages toward a pro-inflammatory phenotype in melanoma, establishing a direct functional link between CH25H activity in the TME and anti-tumor immunity (23). Systematic spatial transcriptomic mapping of HSD3B7 across human solid tumor types remains an important knowledge gap, which we now explicitly acknowledge.

**Figure 2:**
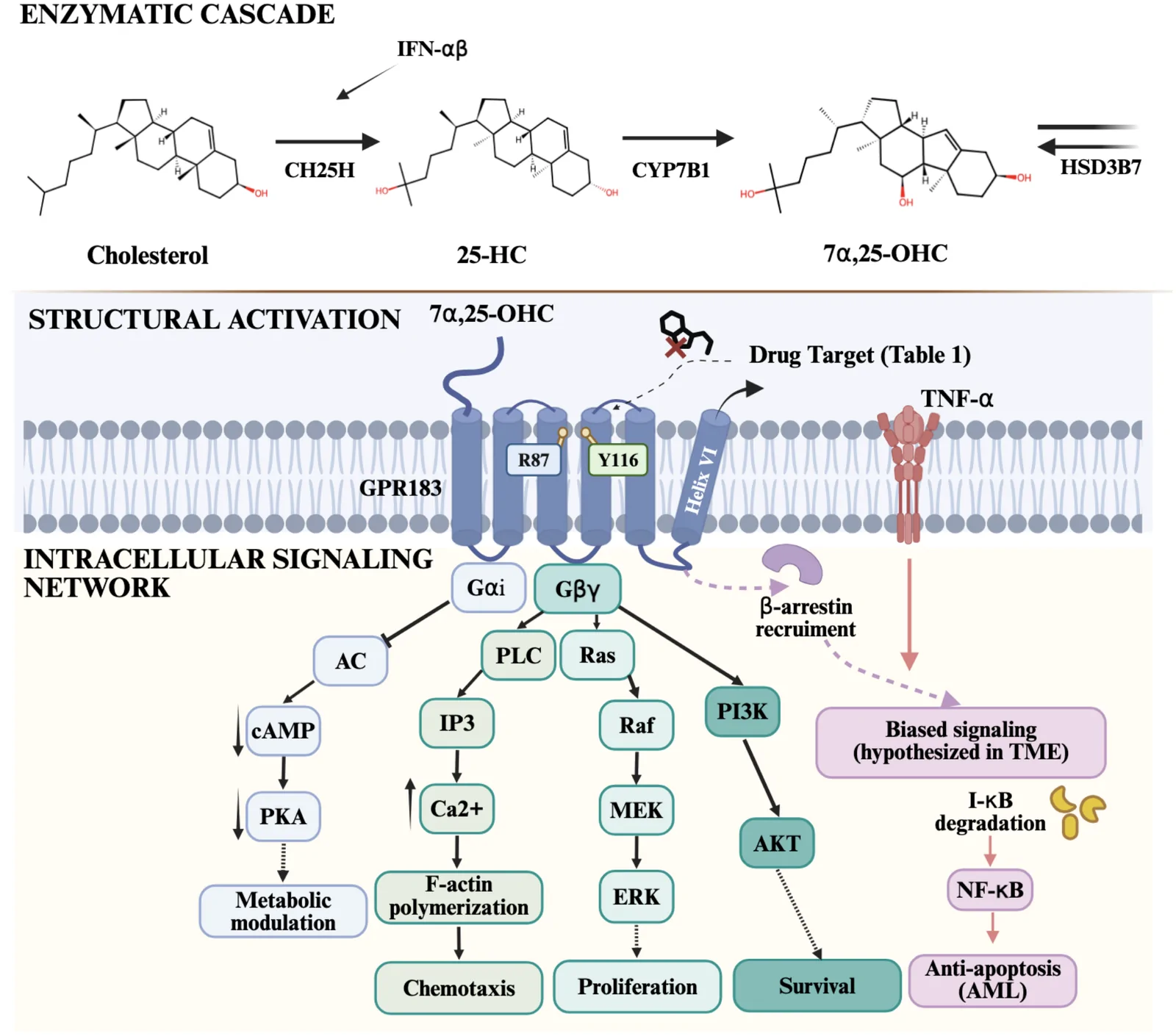
Enzymatic synthesis, structural activation, and intracellular signaling of the GPR183-oxysterol axis. Enzymatic cascade: Sequential synthesis of 7α,25-OHC from cholesterol via CH25H and CYP7B1, countered by HSD3B7-mediated degradation. IFN-α/β signaling transcriptionally induces CH25H expression. Structural activation: 7α,25-OHC anchors the orthosteric pocket via polar interactions with Arg87 and Tyr116, triggering outward displacement of transmembrane helix VI and facilitating Gαi coupling. The defined binding pocket enables structure-guided drug design (**Table 1**). Intracellular signaling network downstream of GPR183 activation. Solid arrows indicate validated pathways; dashed arrows indicate hypothesized mechanisms. 25-HC, 25-hydroxycholesterol; 7α,25-OHC, 7α,25-dihydroxycholesterol; AC, adenylate cyclase; Arg87, arginine 87; cAMP, cyclic adenosine monophosphate; CH25H, cholesterol 25-hydroxylase; CYP7B1, cytochrome P450 family 7 subfamily B member 1; ERK, extracellular signal-regulated kinase; Gαi, inhibitory G protein alpha subunit; Gβγ, G protein beta-gamma dimer; HSD3B7, 3β-hydroxysteroid dehydrogenase type 7; IFN-α/β, type I interferons; IP_b_, inositol trisphosphate; MEK, mitogen-activated protein kinase kinase; NF-κB, nuclear factor kappa B; PI3K, phosphoinositide 3-kinase; PKA, protein kinase A; PLC, phospholipase C; TNF-α, tumor necrosis factor alpha; Tyr116, tyrosine 116.

### 2.2 Structural insights: ligand binding and receptor activation

Recent cryo-electron microscopy (cryo-EM) studies have resolved the GPR183 structure, revealing a canonical seven-transmembrane architecture (24). The 7α,25-OHC ligand is sequestered within a hydrophobic cleft formed by transmembrane helices III, V, and VI (25). High-resolution mapping indicates that the 7α-hydroxyl group interacts with polar residues Arg87 and Tyr116, which anchor the ligand in place (24). Upon binding, the receptor undergoes conformational shifts that propagate to the intracellular loops, marked by an outward displacement of transmembrane helix VI (26), as depicted in the lower panel of **Figure 2**. This structural arrangement accounts for the receptor’s strict specificity for 7α,25-OHC, as other oxysterols lacking the 7α-hydroxyl group fail to stabilize the active conformation (27). Complementing these static structures, computational simulations have elucidated the dynamic activation mechanism of GPR183, revealing a stable partially activated intermediate state that precedes complete receptor activation (28). Structure-based design of GPR183-selective agonists remains an unmet need, although the existing cryo-EM structure and computational tools provide a foundation for future agonist discovery efforts. The translational relevance of these structural findings lies in the potential for structure-guided drug design: the defined binding pocket geometry and the conformational changes at helix VI provide templates for the rational development of both antagonists (targeting hematological malignancies) and agonists (for CAR-T engineering applications), as elaborated in Section 6.1 and **Table 1**.

**Table 1:** GPR183 drug target landscape: therapeutic paradigms, indications, and development status.

Indication	Therapeutic strategy (clinical objective)	Drug target mechanism (molecular pathway)	Agent/Modality	Development stage	Reference
Paradigm I: receptor antagonism (hematological malignancies)					
AML (CN-AML)	Block LSC endosteal anchoring; restore chemosensitivity	GPR183 antagonist → disrupt Gαi signaling → mobilize LSCs from protective niche	Small molecule antagonist (e.g., NIBR189, IC_50_ 11 nM)	Preclinical	(14, 84)
AML (Combination)	Dual BCL-2 + GPR183 blockade to eradicate therapy-resistant LSCs	GPR183 antagonist + Venetoclax → synergistic apoptotic restoration	NIBR189 + Venetoclax	Preclinical (*In vivo* validated)	(15, 36)
ABC-DLBCL	Block constitutive NF-κB survival signaling; disrupt malignant GC retention	GPR183 antagonist → inhibit TNF-α/I-κB/NF-κB axis	NIBR189/structural analogs	Preclinical	(40)
CLL	Prevent GPR183-driven B1a cell clonal expansion	GPR183 antagonist → block oxysterol-mediated B1a proliferation	Targeted screening required	Target validation	(39)
Paradigm II: agonism & microenvironmental modulation (solid tumors)					
Immunologically cold solid tumors	Engineer CAR-T/NK cells to sense 7α,25-OHC; drive deep tumor infiltration	Ectopic GPR183 (agonist mode) → oxysterol-sensing chemotaxis → tumor core penetration	GPR183-CAR-T cells; GPR183-NK cells	Preclinical	(17, 18)
TLS-rich tumors (HNSCC, HCC)	Enhance FRC-derived 7α,25-OHC gradient to promote TLS maturation and ICB response	Endogenous GPR183 (agonist mode) → B cell/DC recruitment → TLS assembly → anti-PD-1 sensitization	Endogenous 7α,25-OHC (FRC-derived); no exogenous agonist available	Biomarker validation	(51, 52)
NSCLC	Preserve endogenous GPR183-mediated metastatic barrier	Endogenous GPR183 → suppress pro-angiogenic signaling → tighten endothelial junctions	Endogenous 7α,25-OHC; no exogenous agent available	Correlative	(16)
Paradigm III: hypothetical role in metabolic inflammation-associated cancers					
MASH-related HCC	Microenvironmental GPR183 blockade to decouple oxysterol-driven myeloid recruitment from oncogenesis	GPR183 antagonist (TME-targeted) → block oxysterol-driven TAM/MDSC chemotaxis	Conceptual target; no validated compound available	Theoretical hypothesis*	(10, 11)

Drug target strategies for the GPR183-oxysterol axis in oncology, organized by therapeutic paradigm. Therapeutic Strategy describes the clinical/translational objective; Drug Target Mechanism details the molecular pathway. Development Stage reflects the most advanced status achieved as of 2026. *Paradigm III Caveat: Direct experimental evidence demonstrating a functional requirement for GPR183 signaling in MASH-to-HCC progression (e.g., GPR183 conditional knockout in MASH-HCC murine models) is currently absent. This paradigm represents an inferential, testable hypothesis based on (a) the established role of 7α,25-OHC in myeloid chemotaxis and (b) the known association between MASLD-associated chronic inflammation and HCC risk. It should not be cited as an established therapeutic paradigm.

7α,25-OHC, 7α,25-dihydroxycholesterol; ABC-DLBCL, activated B-cell-like diffuse large B-cell lymphoma; AML, acute myeloid leukemia; BCL-2, B-cell lymphoma 2; CAR, chimeric antigen receptor; CLL, chronic lymphocytic leukemia; CN-AML, cytogenetically normal AML; FRC, fibroblastic reticular cell; GC, germinal center; GPR183, G protein-coupled receptor 183; HCC, hepatocellular carcinoma; HNSCC, head and neck squamous cell carcinoma; ICB, immune checkpoint blockade; LSC, leukemic stem cell; MASH, metabolic dysfunction-associated steatohepatitis; MDSC, myeloid-derived suppressor cell; NK, natural killer; NSCLC, non-small cell lung cancer; PD-1, programmed cell death protein 1; PI3K, phosphoinositide 3-kinase; TAM, tumor-associated macrophage; Tfh, follicular helper T cell; TLS, tertiary lymphoid structure; TME, tumor microenvironment.

### 2.3 Signal transduction: beyond simple chemotaxis

Beyond chemotaxis, GPR183 activation initiates an intracellular signaling cascade primarily through coupling with the G protein alpha i (Gαi) subunit to inhibit adenylate cyclase and reduce cAMP levels (2). The simultaneous dissociation of the Gβγ dimer activates phospholipase C (PLC), which generates inositol triphosphate (IP3) to trigger calcium release from the endoplasmic reticulum (29). Downstream signaling further integrates through mitogen-activated protein kinase (MAPK) and AKT pathways to regulate cytoskeletal reorganization. These signals are integrated at the cell membrane by Rho GTPases, including Rac1 and Cdc42, which drive filamentous actin (F-actin) polymerization at the leading edge while retracting the trailing uropod (30). These biochemical events convert external metabolic gradients into directional cell movement (30). The specific contribution of Gαi-dependent versus β-arrestin-dependent signaling to distinct cellular outcomes — lymphocyte positioning versus myeloid recruitment, for example — remains unresolved and may represent one determinant of the receptor’s contextual polarity (Section 4.4). These signaling events are summarized in **Figure 2**.

## 3 Hematological malignancies: the intrinsic oncogenic driver

### 3.1 Acute myeloid leukemia: GPR183 as a prognostic sentinel

Hematological malignancies demonstrate a marked intrinsic oncogenic dependency on GPR183, characterized by the frequent overexpression of this receptor in AML blasts (14). This expression profile carries clinical weight in cytogenetically normal AML (CN-AML), a subgroup recognized for its prognostic variability, in which elevated GPR183 levels correlate with reduced overall survival and reduced event-free survival (14). Single-cell RNA sequencing (scRNA-seq) has elucidated the spatial complexity of the leukemic bone marrow niche (31). Within this niche, leukemic stem cells (LSCs) exhibit elevated GPR183 expression (15), which facilitates their anchoring within protective endosteal regions and limits their exposure to the cytotoxic effects of conventional chemotherapy (15, 32). This intrinsic oncogenic dependency positions GPR183 antagonism as the rational therapeutic strategy for AML, as catalogued in **Table 1** (Paradigm I).

### 3.2 The nuclear factor kappa B connection: fueling malignant survival and resistance

Nuclear factor kappa B (NF-κB) signaling contributes to AML stemness and survival (33), and GPR183 activation is linked to this network through downstream signaling axes involving tumor necrosis factor alpha (TNF-α). Receptor activation initiates a signaling cascade involving TNF-α, which facilitates the phosphorylation and proteasomal degradation of inhibitor of kappa B (I-κB) (34). Upon I-κB degradation, NF-κB dimers translocate into the leukemic nucleus to upregulate anti-apoptotic target genes (35), enabling AML blasts to evade programmed cell death and contributing to the marked chemoresistance observed in relapsed patients. The precise molecular connectors between GPR183 activation and NF-κB pathway engagement — whether through direct Gαi-mediated signaling, β-arrestin scaffolding, or indirect autocrine TNF-α amplification — remain to be fully delineated. To address this clinical challenge, preclinical models have demonstrated that small-molecule antagonists of GPR183 synergize with B-cell lymphoma 2 (BCL-2) inhibitors (36). Specifically, the combination of Venetoclax with receptor blockade has been shown to restore apoptotic sensitivity and eradicate therapy-resistant LSC populations *in vivo* (15). This malignant survival signal is further reinforced by an autocrine positive feedback loop, whereby AML cells secrete TNF-α to drive continuous and self-sustaining NF-κB activation (37).

### 3.3 B-cell lymphomas: utilizing the follicular niche for expansion

B-cell lymphomas subvert the physiological navigation functions of GPR183 to facilitate malignant expansion and spatial redistribution. Chronic lymphocytic leukemia (CLL) originates from CD5-positive B cells (38), and transgenic mouse models overexpressing human GPR183 in the B-cell compartment exhibit B1a cell expansion that culminates in late-onset CLL-like malignancies (39). Within the heterogeneous landscape of diffuse large B-cell lymphoma (DLBCL), the receptor’s role varies across molecular subtypes; activated B-cell-like (ABC) DLBCL demonstrates high intrinsic GPR183 expression that correlates with constitutive NF-κB activation and poor clinical outcomes (40). While the dynamic downregulation of GPR183 is required for normal B-cell confinement and maturation within the germinal center (GC) (41), dysregulation of this axis can arrest terminal differentiation, retaining malignant cells within this niche (40). Other aggressive lymphomas engage the axis by upregulating GPR183, thereby facilitating escape from the primary lymph node and promoting systemic dissemination into extrafollicular areas (42). The therapeutic implications of these divergent roles — niche retention versus dissemination — are reflected in the dual pharmacological strategies outlined in **Table 1**.

## 4 Solid tumors: the navigational paradox in the microenvironment

### 4.1 The metastatic barrier: inhibiting angiogenesis and tumor dissemination

Solid tumors engage the GPR183 pathway differently than hematological malignancies, as the receptor demonstrates functional heterogeneity within this context. In non-small cell lung cancer (NSCLC), the receptor exhibits a suppressive mechanism in which GPR183 signaling inhibits visceral metastasis, with high receptor expression correlating with reduced tumor cell invasiveness (43). Mechanistically, receptor activation suppresses pro-angiogenic signaling cascades, tightening endothelial cell junctions and limiting vascular permeability (16). Breast cancer models reveal similar metastasis-limiting functions, in which specific tumor cell subpopulations upregulate the receptor to limit extra-pulmonary spread (44). However, this suppressive capacity is influenced by the lipid metabolic microenvironment; clear cell renal cell carcinoma (ccRCC), for example, depends on altered cholesterol metabolism in which tumors upregulate specific degrading enzymes to modify local oxysterol concentrations (45). While this metabolic rewiring maintains ccRCC viability, the depletion of specific cholesterol metabolites primes the TME for immune evasion, positioning the local oxysterol gradient as a physical barrier against systemic dissemination (46). The spatial coexistence of metastasis-suppressive (endothelial junction tightening, Section 4.1) and immune-suppressive (myeloid recruitment, Section 4.3) functions of the GPR183 axis within the same TME — and how these functions are spatially segregated across peritumoral versus intratumoral compartments — is examined in Section 4.4.

### 4.2 Tertiary lymphoid structures: intra-tumoral immune organization

The spatial organization of immune cells determines patient prognosis, as illustrated by tertiary lymphoid structures (TLS), which represent ectopic lymphoid aggregates within non-lymphoid tissues (47). Mature TLS formation correlates with favorable clinical outcomes and predicts the efficacy of immune checkpoint blockade (ICB) therapy across multiple solid tumors (48), as depicted in the anti-tumor arm of **Figure 3**. GPR183 serves as a primary driver of this architectural organization by directing B cell and dendritic cell (DC) positioning within the tumor stroma (49). During TLS assembly, fibroblastic reticular cells (FRCs) secrete chemokines that support structural organization, and B cells utilize localized 7α,25-OHC gradients to migrate toward these fibroblastic zones and initiate germinal center formation (50). The FRC-derived oxysterol gradient that governs this process is illustrated in **Figure 3A**. This spatial dynamic is demonstrated in head and neck squamous cell carcinoma (HNSCC), a malignancy exhibiting high TLS density in which mature structures predict successful anti-programmed cell death protein 1 (anti-PD-1) responses (51). In hepatocellular carcinoma (HCC), patients receiving neoadjuvant ICB demonstrate enriched GPR183 signatures within TLS-rich regions (52), indicating that the receptor contributes to the establishment of an organized immune niche that enables local antigen presentation and T cell priming (53). The association between GPR183-driven TLS maturation, CD8+ T cell infiltration density, and ICB response rates across tumor types is catalogued in **Table 1** (Paradigm II).

**Figure 3:**
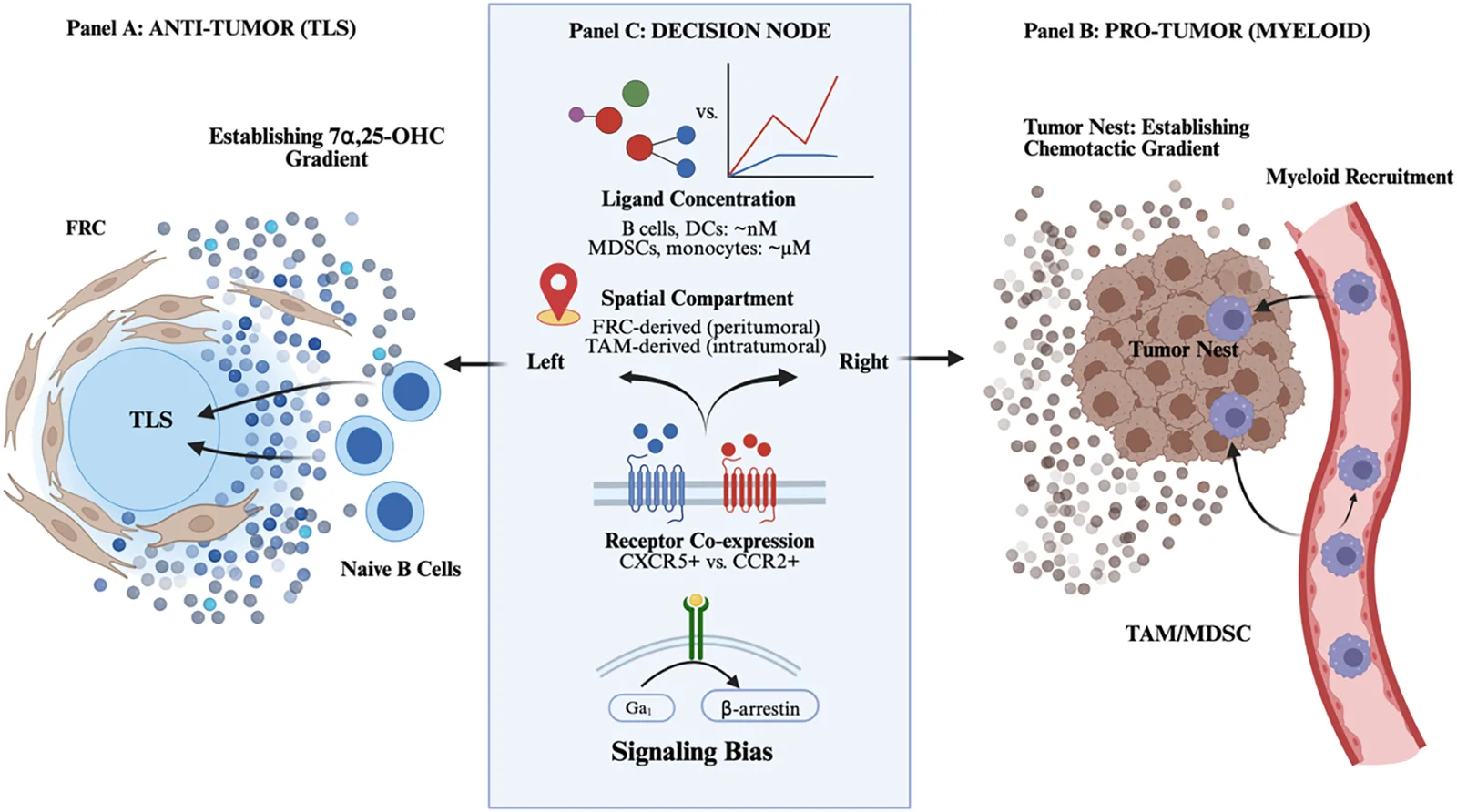
The paradoxical roles of GPR183 signaling in the solid tumor microenvironment. **(A)** Anti-tumor immunity: Physiological 7α,25-OHC gradients derived from FRCs recruit GPR183+ B cells, DCs, and Tfh cells to assemble mature TLS. **(B)** Pro-tumorigenic reprogramming: Supraphysiological gradients from tumor cells and TAMs aberrantly recruit monocytes and MDSCs, establishing an immunosuppressive niche. **(C)** Decision Node: The molecular switch determining TLS assembly versus myeloid recruitment is dictated by four core determinants: ligand concentration thresholds (B cells, DCs: ~nM; MDSCs, monocytes: ~μM), spatial compartmentalization (FRC-derived peritumoral vs. TAM-derived intratumoral), receptor co-expression profiles (e.g., CXCR5+ vs. CCR2+), and downstream signaling bias. 7α,25-OHC, 7α,25-dihydroxycholesterol; CCR2, C-C chemokine receptor type 2; CCL19/21, C-C motif chemokine ligand 19/21; CH25H, cholesterol 25-hydroxylase; CXCR5, C-X-C chemokine receptor type 5; CYP7B1, cytochrome P450 family 7 subfamily B member 1; DC, dendritic cell; FRC, fibroblastic reticular cell; GPR183, G protein-coupled receptor 183; IL-10, interleukin-10; iNOS, inducible nitric oxide synthase; MDSC, myeloid-derived suppressor cell; TAM, tumor-associated macrophage; Tfh, T follicular helper cell; TGF-β, transforming growth factor beta; TLS, tertiary lymphoid structure; TME, tumor microenvironment.

### 4.3 Immunosuppressive myeloid cell recruitment

The GPR183 navigational system presents a distinct paradox within the TME: the same oxysterol gradient is capable of recruiting immunosuppressive cell populations. Inflammatory conditions within the TME enhance oxysterol production; tumor-associated macrophages (TAMs) synthesize 25-HC and 7α,25-OHC in response to chronic inflammatory cues (12, 22), establishing a pathological gradient that recruits myeloid-derived suppressor cells (MDSCs) and additional TAM precursors, as shown in the pro-tumor arm of **Figure 3B**. Tfh cells express active GPR183 and migrate along the gradient to interact with DCs, a recruitment process that modulates the local immune landscape and influences anti-tumor immunity within TLS-rich regions (54). In breast cancer, metastases depend on a systemic lipid-associated macrophage niche, in which CD74-positive macrophages accumulate at metastatic sites to drive T cell suppression (55). Pan-cancer analyses confirm this dual immunoregulatory role, demonstrating that the net clinical outcome of the GPR183 axis depends on the dominant recruited cell type (56). The molecular determinants that govern whether a given 7α,25-OHC gradient drives TLS assembly (**Figure 3A**) versus myeloid-mediated immunosuppression (**Figure 3B**) are systematically examined in Section 4.4.

### 4.4 Determinants of contextual polarity: what governs the anti-tumor versus pro-tumor outcome of the GPR183 axis?

A central mechanistic question emerges from the opposing functions described in Sections 4.2 and 4.3: what factors determine whether a localized 7α,25-OHC gradient drives protective TLS formation or pathogenic myeloid recruitment (**Figure 3**)? We propose four non-mutually exclusive determinants — schematized as a decision node in **Figure 3C** — that may govern this contextual polarity. These four determinants are proposed as a conceptual framework; each remains hypothetical and requires systematic experimental validation.

#### 4.4.1 Ligand concentration thresholds

Differential sensitivity of lymphocyte versus myeloid populations to 7α,25-OHC concentration may determine recruitment outcomes. B cells and DCs respond to 7α,25-OHC at concentrations in the nanomolar range *in vitro*, consistent with the shallow gradients generated by FRCs in lymphoid tissues (57). In contrast, the supraphysiological oxysterol concentrations produced within hypoxic and inflamed tumor cores (22) may preferentially engage myeloid populations expressing higher levels of the receptor or exhibiting distinct dose-response coupling to downstream effectors. Quantitative comparisons of GPR183 surface density and ligand sensitivity across B cells, DCs, Tfh cells, monocytes, and MDSCs have not been systematically reported and represent a critical experimental priority. Whether these concentration-dependent effects operate *in vivo*, however, has not been examined.

#### 4.4.2 Spatial compartmentalization of oxysterol production

The cellular source of 7α,25-OHC may qualitatively differ between the peritumoral stroma and the intratumoral core. FRC-derived oxysterol, generated through regulated CH25H/CYP7B1 expression in the tumor stroma, co-localizes with homeostatic chemokines (CCL19, CCL21) that support lymphoid organization (4, 50). By contrast, tumor cell-autonomous or TAM-derived oxysterol production within the hypoxic core co-occurs with immunosuppressive mediators including interleukin-10 (IL-10) and transforming growth factor beta (TGF-β) (13). Thus, GPR183-expressing cells arriving in the peritumoral stroma encounter a lymphoid-organizing signaling milieu, whereas those entering the tumor core encounter an immunosuppressive signaling milieu, even though both are recruited by the same oxysterol species. The spatial distribution of CH25H and CYP7B1 expression across these compartments has been partially characterized in lymphoid tissues but has been partially addressed by recent spatial transcriptomic data in melanoma (23), although systematic mapping across human solid tumor types remains an important knowledge gap. Direct evidence causally linking oxysterol source to recruitment outcome is lacking.

#### 4.4.3 Receptor co-expression patterns

The functional outcome of GPR183 engagement may depend on the co-expression of additional chemokine receptors that modify the cell’s ultimate tissue destination and functional polarization. GPR183+CXCR5+ B cells are directed toward follicular niches and participate in GC reactions and TLS assembly (41, 58). Conversely, GPR183+CCR2+ monocytes are directed toward inflammatory and tumor sites, where they differentiate into TAMs (59). GPR183+CXCR3+ T cells may be recruited to distinct microanatomical sites compared with GPR183+CXCR4+ T cells (60). GPR183+CXCR3+ T cells may be preferentially directed toward peritumoral regions enriched in CXCL9/CXCL10, where effector function is preserved, while GPR183+CXCR4+ T cells may be retained in stromal zones expressing CXCL12, potentially adopting an exhausted or regulatory phenotype. This differential microanatomical targeting — effector-permissive versus stromal-retentive — may represent an additional layer of contextual polarity at the T cell level that has not been experimentally dissected. The combinatorial expression of these receptors on individual cells — and how heterodimerization between GPR183 and CXCR5 alters ligand sensitivity or signaling bias (60, 61) — may constitute the molecular basis for cell-type-specific responses to identical oxysterol gradients. Perturbation experiments that isolate individual receptor contributions to GPR183-dependent trafficking have yet to be performed.

#### 4.4.4 Downstream signaling bias

GPR183, like other GPCRs, can signal through both Gαi-dependent and β-arrestin-dependent pathways. Biased agonism — in which different ligands or different ligand concentrations preferentially activate one pathway over the other — has been documented for several chemokine receptors 25. Whether the FRC-derived 7α,25-OHC gradient (TLS-promoting) and the TAM-derived 7α,25-OHC gradient (MDSC-recruiting) differ in their capacity to induce Gαi versus β-arrestin signaling has not been investigated. Ligand concentration, the presence of allosteric modulators within the TME, and receptor heterodimerization status may all influence signaling bias and thereby determine cellular outcome. Direct interrogation of ligand- or concentration-dependent signaling bias at GPR183 in primary immune cells remains an outstanding experimental priority.

These four determinants are not independent; they are likely to interact hierarchically. For instance, spatial compartmentalization (source) may dictate local ligand concentration (dose), which in turn triggers biased signaling (pathway) in a cell-type-specific manner (co-receptor context). Systematic experimental dissection of these interacting variables — through spatial transcriptomics of CH25H/CYP7B1 expression, quantitative measurements of intratumoral 7α,25-OHC concentrations by mass spectrometry imaging, and single-cell resolution analyses of GPR183 co-receptor expression — will be required to resolve the central paradox of the GPR183 axis in solid tumors.

From a translational standpoint, these four determinants differ markedly in experimental tractability and clinical relevance, which suggests a natural prioritization for future investigation. Spatial compartmentalization (Section 4.4.2) represents the most tractable and clinically informative entry point: established spatial transcriptomic platforms (e.g., Visium, Xenium, MERFISH) can directly map CH25H and CYP7B1 expression across TLS, peritumoral stroma, and intratumoral core in archival human tumor specimens, providing an immediate readout of whether the oxysterol source predicts immune outcome, while simultaneously informing patient stratification and TLS-guided therapy. Receptor co-expression patterns (Section 4.4.3) are likewise tractable using single-cell and spatial multi-omics (e.g., CITE-seq), which resolve the combinatorial receptor repertoire on individual GPR183+ cells. Ligand concentration thresholds (Section 4.4.1) require quantitative mass spectrometry imaging of 7α,25-OHC, which is technically feasible but constrained by spatial resolution and analyte standardization. Downstream signaling bias (Section 4.4.4) is the most mechanistically fundamental yet experimentally demanding determinant, requiring conformational biosensors or pathway-specific reporters that have only recently become available (26). We therefore propose spatial compartmentalization as the priority entry point for resolving contextual polarity — being simultaneously the most experimentally accessible and the most directly relevant to clinical translation — with the remaining determinants addressed in sequence as enabling technologies mature.

## 5 The frontier: synthetic immunology and CAR-T cell engineering

### 5.1 The infiltration dilemma: why conventional cell therapies fail in solid tumors?

While adoptive cell therapies, particularly CAR-T cells, have demonstrated efficacy in hematological malignancies (62), their application against solid tumors remains constrained by the dense and immunosuppressive stromal architecture (63). The physical and biochemical barrier restricts the spatial penetration of therapeutic effector cells (64) and subjects them to metabolic starvation and exhaustion (65), creating an infiltration dilemma. Dysregulated tumor angiogenesis and disordered chemokine production act as biological barriers (66), while a dense extracellular matrix dominated by cancer-associated fibroblasts (CAFs) further excludes immune infiltrates (67). Consequently, conventional cell therapies frequently fail to navigate the TME topography (68), resulting in superficial localization at the tumor periphery and suboptimal tumor eradication (69). This physical exclusion establishes a clinical requirement for engineered homing strategies capable of penetrating the stromal barrier (70), as schematized in **Figure 4**.

**Figure 4:**
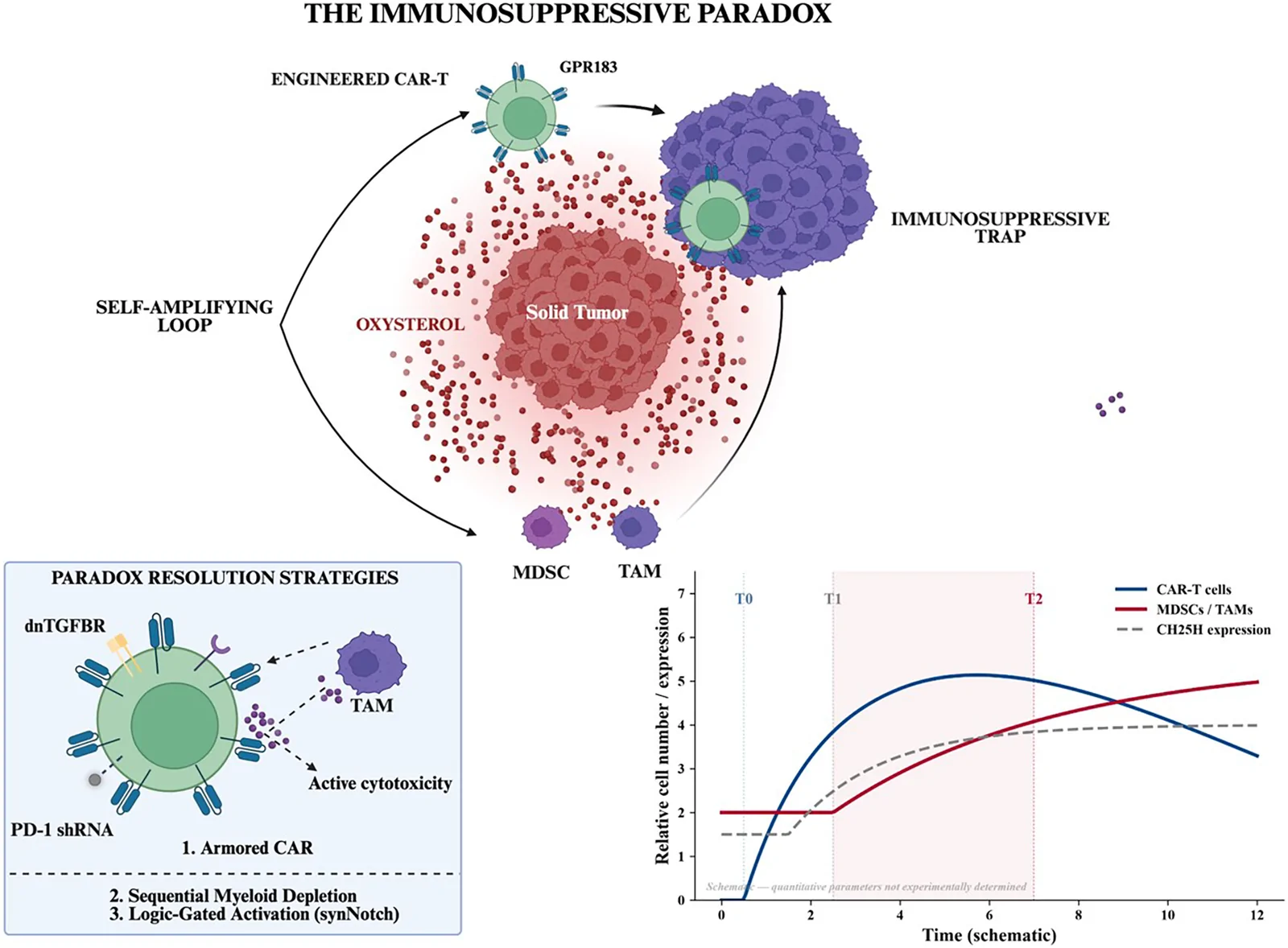
Synthetic immunology rewiring of CAR-T cells via the GPR183-oxysterol axis. Top: GPR183-engineered CAR-T cells sense 7α,25-OHC gradients, infiltrate solid tumors, and trigger CH25H upregulation upon tumor lysis. Bottom left: Resolution strategies. Bottom right: Schematic dynamic equilibrium of CAR-T cells, MDSCs, and CH25H expression over time. T0, CAR-T infusion; T1, CH25H upregulation; T2, MDSC accumulation exceeds CAR-T cells. Quantitative parameters are not experimentally determined. 7α,25-OHC, 7α,25-dihydroxycholesterol; CAF, cancer-associated fibroblast; CAR, chimeric antigen receptor; CH25H, cholesterol 25-hydroxylase; GPR183, G protein-coupled receptor 183; IFN-γ, interferon gamma; MDSC, myeloid-derived suppressor cell; PD-1, programmed cell death protein 1; shRNA, short hairpin RNA; TAM, tumor-associated macrophage; TGF-β, transforming growth factor beta; TME, tumor microenvironment.

### 5.2 Metabolite sensing via GPR183

To overcome restrictive stromal barriers, synthetic immunology has developed the concept of metabolite sensing by equipping engineered effector cells with biochemical sensors (71, 72). Building upon the precedent of rewiring T cells with exogenous chemokine receptors to enhance tumor localization (73, 74), current strategies target tumor-specific metabolic gradients. By rewiring CAR-T cells to ectopically co-express GPR183, therapeutic cells are modified to detect and track the endogenous 7α,25-OHC oxysterol gradients established by TAMs (18), as illustrated in **Figure 4**. This engineering approach converts a metabolic pathway co-opted by solid tumors for immune evasion into a targeted navigational tool (75). Such microenvironment remodeling enables modified CAR-T cells to bypass physical exclusion, migrate toward the tumor core (76), and achieve deep intra-tumoral tissue penetration (77).

The strategy of directing CAR-T cells toward 7α,25-OHC gradients must contend with the immunological paradox identified in Sections 4.3 and 4.4: the same gradient that guides engineered CAR-T cells into the tumor also recruits immunosuppressive TAMs and MDSCs. The terminal destination of GPR183-guided CAR-T cells is therefore predicted to be a microenvironment enriched for suppressive myeloid populations capable of inactivating T cell effector function. This is not a theoretical concern; it follows directly from the established biology described in Section 4.3. We propose three complementary strategies to address this paradox. First, armored CAR engineering: GPR183-expressing CAR-T cells can be co-engineered with resistance modules that neutralize MDSC- and TAM-derived suppressive signals. Examples include co-expression of a dominant-negative TGF-β receptor to block TGF-β-mediated suppression, PD-1 short hairpin RNA (shRNA) to prevent PD-L1/PD-L2-mediated exhaustion, or a chimeric cytokine receptor that converts immunosuppressive IL-4 into a T cell-stimulatory signal (78). Second, sequential combination therapy: pharmacological or biological depletion of MDSCs and TAMs — through colony-stimulating factor 1 receptor (CSF1R) inhibition, CCL2/CCR2 axis blockade, or CD11b-targeted depletion — prior to GPR183-CAR-T cell infusion may temporarily clear the immunosuppressive niche, creating a permissive window for CAR-T infiltration and effector function. Third, engineering conditional activation: synthetic Notch (synNotch) or logic-gated CAR circuits can be designed such that GPR183-mediated migration and CAR-mediated cytotoxicity are only activated upon encountering a tumor-specific antigen, thereby restricting effector function to the tumor core while sparing normal tissues. Whether the CAR-T cells described in recent studies (17, 18) retain cytotoxic function within MDSC-rich microenvironments has not been directly demonstrated and represents a critical experimental priority for this field.

Each of these strategies, however, carries distinct feasibility and safety considerations that warrant explicit discussion. Armored CAR engineering with dominant-negative TGF-β receptors or PD-1 shRNA has shown efficacy in preclinical solid-tumor models (78), but constitutive TGF-β blockade may impair regulatory T cell function and peripheral tolerance (79), and persistent PD-1 knockdown risks unrestrained T cell activation and autoimmune toxicity (80); inducible or tumor-restricted expression systems would mitigate, though not eliminate, this risk. Sequential myeloid depletion through CSF1R inhibition or CCL2/CCR2 blockade is complicated by the physiological roles of tissue-resident macrophages — CSF1R inhibition has been associated with dose-limiting hepatotoxicity and disrupted Kupffer cell homeostasis (81), whereas CCR2 blockade may impair protective monocyte trafficking during infection (82) — and by the difficulty of aligning the depletion window with CAR-T infusion. Logic-gated (synNotch) circuits offer the greatest tumor specificity but introduce additional engineering complexity, potential attenuation of signal strength, and susceptibility to antigen-loss escape. A balanced translational perspective therefore favors a staged approach: empirical validation of armored modules under inducible control as the near-term priority, with logic-gated specificity incorporated as engineering platforms mature, and myeloid-depleting combinations reserved for settings in which target antigens are validated and the expected toxicity profile is favorable.

### 5.3 Positive feedback loops: a self-sustaining infiltration strategy

Building upon enhanced navigational capacity, the integration of GPR183 signaling aligns with the paradigm of armored adoptive cell therapies (78), potentially establishing a self-amplifying recruitment loop. As initial GPR183-engineered effector cells infiltrate the TME and initiate localized tumor cell lysis, the resulting inflammatory milieu can stimulate CH25H expression in resident myeloid cells (12), thereby amplifying local oxysterol production and facilitating sustained secondary recruitment (18). This dynamic metabolic shift establishes a self-sustaining cycle of effector cell recruitment, analogous to engineered circuits that secrete pro-inflammatory cytokines to sustain immune responses (78, 83). This self-sustaining infiltration strategy, illustrated in **Figure 4**, provides a mechanism to maintain long-term anti-tumor immunosurveillance and convert immunologically inactive tumors into responsive therapeutic targets.

The positive feedback model, however, carries an inherent amplification risk: the same CH25H upregulation that recruits additional CAR-T cells would also recruit additional MDSCs and TAMs, as indicated by the dual pathway illustrated in **Figure 3**. Unless the engineered CAR-T cells possess the resistance mechanisms discussed in Section 5.2, each cycle of the feedback loop would increase the ratio of suppressive myeloid cells to effector T cells at the tumor site. The net therapeutic outcome therefore depends on whether the armored resistance modules engineered into the CAR-T cells can outpace the concurrent amplification of local immunosuppression. Quantitative modeling of this dynamic — incorporating parameters such as CH25H induction kinetics, CAR-T proliferation rate, MDSC recruitment rate, and the degree of armored resistance — would inform the rational design of this therapeutic strategy.

## 6 Clinical implications and translational perspectives

### 6.1 Precision targeting: context-dependent antagonists versus agonists

The transition of the GPR183-oxysterol axis from immunological discovery to clinical oncology necessitates a pharmacological approach dictated by the malignant context, as systematically delineated in **Table 1** (84). Three distinct therapeutic paradigms have emerged, and their molecular rationales and translational statuses are summarized in **Table 1**.

Paradigm I — Receptor Antagonism in Hematological Malignancies (**Table 1**). In AML, where intrinsic GPR183 overexpression drives malignant survival and therapy resistance, therapeutic intervention requires receptor blockade 76,77. Medicinal chemistry efforts have yielded small-molecule antagonists including NIBR189, which demonstrates nanomolar potency in disrupting oxysterol-induced signaling in preclinical models 76. The combination of GPR183 antagonism with BCL-2 inhibition (Venetoclax) has shown efficacy in pre-clinical AML models by restoring apoptotic sensitivity in LSCs 15,34. In B-cell lymphomas, the therapeutic objective depends on the molecular subtype: receptor blockade may prevent malignant niche retention in ABC-DLBCL, whereas in lymphomas utilizing GPR183 for dissemination, antagonism may reduce systemic spread 38,40. The clinical development status of GPR183 antagonists — currently at the preclinical stage for all listed indications — is detailed in **Table 1**. Structure-based computational approaches have accelerated GPR183 drug discovery: a chemometrics-driven multilayered lead prioritization strategy integrating pharmacophore modeling, molecular docking, and MD simulations has identified novel benzothiazole-based GPR183 antagonists with sub-micromolar potency (85). In parallel, medicinal chemistry efforts have yielded small-molecule antagonists including NIBR189 and several benzothiazole derivatives with improved selectivity profiles, currently undergoing lead optimization.

Paradigm II — Receptor Agonism or Microenvironmental Modulation in Solid Tumors (**Table 1**). In solid tumors characterized by dense stromal barriers and immune exclusion, the therapeutic objective shifts toward enhancing effector cell infiltration (86, 87). In immunologically cold microenvironments, synthetic receptor agonists may empower engineered T cells to track tumoral oxysterol gradients (88). In TLS-rich tumors, pharmacological enhancement of the endogenous GPR183 axis — through stabilization of FRC-derived 7α,25-OHC gradients — may promote TLS maturation and improve ICB response rates, as suggested by the association between GPR183 signatures and anti-PD-1 efficacy in HNSCC and HCC (**Table 1**) (51, 52). In NSCLC, preservation of the endogenous GPR183-mediated metastatic barrier represents an additional therapeutic objective (16).

Paradigm III — Hypothetical Role in Metabolic Inflammation-Associated Cancers (**Table 1**). A third emerging paradigm targets malignancies arising in the context of metabolic dysfunction, where dysregulated lipid metabolism recruits immunosuppressive macrophages through the GPR183 axis. In metabolic dysfunction-associated steatohepatitis (MASH)-related hepatocarcinogenesis, the hypothesis is that chronic oxysterol production in the steatotic and inflamed liver establishes a chemotactic gradient that recruits GPR183-expressing myeloid cells, contributing to an immunosuppressive pre-malignant niche (10, 11). Microenvironmental blockade of the GPR183 axis in this context represents a translational frontier to decouple metabolic dysfunction from oncogenesis. However, direct experimental evidence demonstrating a functional requirement for GPR183 signaling in MASH-to-HCC progression is currently lacking; this paradigm remains a testable hypothesis. Accordingly, we frame the MASH-HCC link strictly as a testable hypothesis — an inference grounded in the established role of oxysterols in myeloid chemotaxis and the known association between MASLD-related chronic inflammation and HCC risk — rather than as an established mechanistic pathway. The evidence gap is explicitly noted in **Table 1**.

Precision targeting in oncology therefore requires a bidirectional strategy: deploying antagonists to dismantle oncogenic drivers in liquid tumors, and deploying agonists or gradient-enhancing strategies to promote effector cell infiltration in solid tumors. The distinct pharmacological imperatives and their evidentiary foundations are catalogued by tumor type, therapeutic modality, and clinical development status in **Table 1**.

#### 6.1.1 Translational challenges

The transition of GPR183-targeted therapies from preclinical investigation to clinical application faces several specific obstacles that warrant explicit consideration. First, CNS safety of NIBR189 and related antagonists: The blood-brain barrier (BBB) permeability of NIBR189 has not been systematically characterized. GPR183 is expressed in the central nervous system, where it contributes to neuropathic pain processing (89). Systemic administration of brain-penetrant GPR183 antagonists could therefore interfere with the physiological functions of GPR183 in the CNS, potentially producing neurological adverse effects that would require careful monitoring in early-phase clinical trials.

Second, intestinal immune homeostasis: GPR183 is highly expressed in the small intestine and colon, where the GPR183-oxysterol axis directs immune cell positioning within intestinal lymphoid follicles and contributes to mucosal immune surveillance. Genetic deletion or pharmacological blockade of GPR183 has been shown to exacerbate intestinal inflammation in murine models (7, 8). Systemic GPR183 antagonism may therefore compromise intestinal immune homeostasis, and the risk of inflammatory bowel disease (IBD)-like adverse events should be prospectively evaluated.

Third, the agonist development gap: Unlike antagonists, for which NIBR189 and several benzothiazole-based compounds have entered lead optimization, no GPR183-specific agonists have advanced to preclinical development. The pharmacokinetic properties, toxicity profiles, and receptor selectivity of synthetic GPR183 agonists remain completely unknown. The development of biased agonists that selectively activate Gαi-mediated chemotaxis without engaging β-arrestin-mediated pathways represents an attractive but experimentally unrealized strategy.

Fourth, the CAR-T immunosuppressive paradox: As discussed in Section 5.2, GPR183-guided CAR-T cells are directed into milieux rich in 7α,25-OHC, the same chemotactic environment that recruits MDSCs and immunosuppressive myeloid populations. This creates a self-amplifying cycle: CAR-T-mediated tumor lysis triggers inflammatory CH25H upregulation, which increases local 7α,25-OHC production, which in turn recruits additional CAR-T cells but also additional MDSCs. Each successive cycle may increase the ratio of suppressive myeloid cells to effector T cells within the tumor, potentially undermining the durability of CAR-T responses. Quantitative modeling of this dynamic equilibrium has not been performed and would inform the rational design of combination regimens incorporating myeloid checkpoint blockade. The immunosuppressive paradox has not been experimentally tested; preclinical demonstration of CAR-T effector function within MDSC-rich TMEs is a prerequisite for clinical translation.

### 6.2 Biomarker potential in the era of personalized immunotherapy

In the era of personalized medicine, GPR183 profiling presents an opportunity to identify patients who may derive maximal benefit from targeted immunotherapeutic regimens (4, 89). Transcriptomic analyses have demonstrated an association between GPR183 expression and the regulation of inflammatory processes, as well as a correlation with overall survival across multiple malignancies (43, 86). Beyond prognosis, the spatial architecture of the receptor serves as a marker for mature TLS formation and intra-tumoral immune infiltration (87, 90). Clinical data from 2024–2025 indicate that GPR183 expression patterns may correlate with responsiveness to ICB therapies, particularly in patients in whom the receptor directs the recruitment of DCs and Tfh cells (50, 90). The integration of GPR183 expression with TLS maturation status, CD8+ T cell density, and PD-L1 combined positive score (CPS) as a composite biomarker panel — rather than reliance on any single parameter — may improve predictive accuracy for ICB response stratification. The biomarker associations across tumor types — including GPR183 expression correlations with immune infiltration, survival outcomes, and TLS maturation status — are discussed within each tumor-type paragraph in Sections 4.2–4.3 and summarized within the Paradigm II entries of **Table 1**.

### 6.3 Unresolved questions: navigating the future research directions

Several mechanistic questions remain that constrain the clinical translation of GPR183-targeted therapies (91). A major unresolved paradigm involves the structural heterodimerization of GPR183 with other chemokine receptors, including C-X-C chemokine receptor type 5 (CXCR5), which may modify the interpretation of localized chemotactic signaling within the TME (61), as discussed in Section 4.4. The systemic consequences of pharmacological modulation require evaluation to balance anti-tumor efficacy against potential off-target effects, including disruptions in basal neuropathic pain regulation (89) and endothelial senescence (88). The functional role of GPR183 in MASH-related hepatocarcinogenesis — whether the receptor actively contributes to malignant transformation in the steatotic liver microenvironment, or whether its involvement is limited to the associated inflammatory state — remains an open question requiring direct experimental investigation. Furthermore, the immunosuppressive paradox in GPR183-guided CAR-T therapy (Section 5.2) requires resolution through preclinical studies that directly measure CAR-T effector function within MDSC-rich microenvironments. Resolving these questions through high-resolution spatial multi-omics, lipidomic profiling, and quantitative *in vivo* models will be required for steering the GPR183 axis toward clinical oncology (88). With respect to the MASH-HCC paradigm specifically, several prioritized experiments are needed: (i) longitudinal single-cell profiling of CH25H/CYP7B1/HSD3B7 expression across the MASH-to-HCC transition in murine models and human biopsies; (ii) functional validation using hepatocyte-specific CH25H knockout mice in established MASH-HCC models; (iii) spatial transcriptomic mapping of the GPR183-oxysterol axis across the full histologic spectrum from steatosis to steatohepatitis to HCC; and (iv) correlation of serum oxysterol profiles with GPR183-dependent immune parameters in MASH-HCC patient cohorts. It must be underscored that direct functional evidence linking GPR183 signaling to MASH-associated hepatocarcinogenesis remains to be established, and this paradigm currently represents a theoretical hypothesis grounded in the known immunomodulatory functions of the GPR183-oxysterol axis in metabolic inflammation.

Beyond the chemotactic and signaling functions discussed above, the GPR183-oxysterol axis may also interface with the broader epigenetic and transcriptional regulatory landscape of the TME. Oxysterols serve as endogenous ligands for liver X receptors (LXRs), nuclear receptors that govern inflammatory and metabolic gene programs and can recruit chromatin-modifying complexes, thereby modulating the epigenetic landscape of both tumor and immune cells (92). Emerging evidence indicates that epigenetic and transcriptional dysregulation can promote metastasis through specific downstream effectors — for example, loss of the histone methyltransferases KMT2C/D drives breast cancer brain metastasis through KDM6A-mediated epigenetic remodeling (93). Whether oxysterol signaling through GPR183 and LXRs exerts indirect effects on chromatin remodeling and transcriptional reprogramming within the TME — and whether targeting this axis might therefore carry broader epigenetic consequences — remains an open question that warrants dedicated investigation.

The transition of GPR183 from a chemotactic sensor to an actionable clinical target depends on deciphering its dual nature within the TME. The receptor functions not merely as a passive participant in immune cell trafficking but as a central mediator that translates systemic metabolic fluctuations — specifically, oxysterol gradients — into spatial immunological outcomes. By integrating context-dependent pharmacological modulation with precision biomarker profiling, and by addressing the unresolved structural and functional complexities enumerated above, future therapeutic designs may fully harness the GPR183 axis. This integrated approach may enable the development of next-generation immunotherapies that reprogram the metabolic-immune ecosystem to favor anti-tumor immunity.

## 7 Conclusion

The biology of the GPR183-oxysterol axis reveals a connection between lipid metabolism and immune organization within the TME. Rather than functioning solely as a chemotactic receptor, GPR183 coordinates immune cell positioning and inflammatory architecture across diverse pathological settings, including hematological malignancies, solid tumors, and metabolic-inflammatory conditions. As discussed throughout this review, therapeutic targeting of this pathway requires context-dependent strategies: receptor antagonism in hematological malignancies and modulation of immune cell trafficking and TLS assembly in solid tumors. The central paradox of the axis — that identical 7α,25-OHC gradients can drive either protective TLS formation or immunosuppressive myeloid recruitment — must be resolved through systematic investigation of the four candidate determinants identified in Section 4.4. Similarly, the immunosuppressive paradox inherent in GPR183-guided CAR-T therapy (Section 5.2) requires direct experimental attention before clinical translation can proceed.

Future clinical translation of GPR183-targeted therapies will depend on advances in spatial multi-omics, quantitative lipidomic profiling, and structure-guided drug development. A deeper understanding of how oxysterol signaling shapes immune cell dynamics — including the concentration thresholds, spatial compartmentalization, co-receptor expression patterns, and signaling bias mechanisms that govern contextual polarity — may facilitate the rational design of therapies capable of remodeling the TME. Continued investigation of the GPR183 axis may yield new opportunities for precision immuno-oncology and metabolism-oriented cancer therapy.

## Glossary

25-HC: 25-hydroxycholesterol
7α,25-OHC: 7α,25-dihydroxycholesterol
ABC-DLBCL: activated B-cell-like diffuse large B-cell lymphoma
AML: acute myeloid leukemia
Arg87: arginine 87
BCL-2: B-cell lymphoma 2
CAF: cancer-associated fibroblast
cAMP: cyclic adenosine monophosphate
CAR-T: chimeric antigen receptor T (cell)
CCL19/21: C-C motif chemokine ligand 19/21
CH25H: cholesterol 25-hydroxylase
CLL: chronic lymphocytic leukemia
CN-AML: cytogenetically normal acute myeloid leukemia
CXCR5: C-X-C motif chemokine receptor 5
CCR2: C-C motif chemokine receptor 2
CYP7B1: cytochrome P450 family 7 subfamily B member 1
DC: dendritic cell
dnTGFBR: dominant-negative transforming growth factor beta receptor
FRC: fibroblastic reticular cell
Gαi: inhibitory G protein alpha subunit
GC: germinal center
GPR183: G protein-coupled receptor 183
HCC: hepatocellular carcinoma
HNSCC: head and neck squamous cell carcinoma
HSD3B7: 3β-hydroxysteroid dehydrogenase type 7
ICB: immune checkpoint blockade
IFN-α/β: type I interferons
IFN-γ: interferon gamma
IL-10: interleukin-10
iNOS: inducible nitric oxide synthase
LSC: leukemic stem cell
MASH: metabolic dysfunction-associated steatohepatitis
MDSC: myeloid-derived suppressor cell
NF-κB: nuclear factor kappa light chain enhancer of activated B cells
NK: natural killer (cell)
NSCLC: non-small cell lung cancer
PD-1: programmed cell death protein 1
PI3K: phosphoinositide 3-kinase
shRNA: short hairpin RNA
synNotch: synthetic Notch (receptor)
TAM: tumor-associated macrophage
Tfh: follicular helper T (cell)
TGF-β: transforming growth factor beta
TLS: tertiary lymphoid structure
TME: tumor microenvironment
TNF-α: tumor necrosis factor alpha
Tyr116: tyrosine 116
